# Barriers and facilitators of pediatric shared decision-making: a systematic review

**DOI:** 10.1186/s13012-018-0851-5

**Published:** 2019-01-18

**Authors:** Laura Boland, Ian D. Graham, France Légaré, Krystina Lewis, Janet Jull, Allyson Shephard, Margaret L. Lawson, Alexandra Davis, Audrey Yameogo, Dawn Stacey

**Affiliations:** 1 Faculty of Health Sciences, University of Ottawa, 540 King Edward Avenue, Ottawa, ON K1N 6N5 Canada; 2 Ottawa Hospital Research Institute, 501 Smyth Road, Ottawa, ON K1H 8L6 Canada; 3 School of Epidemiology and Public Health, Faculty of Medicine, University of Ottawa, 307D-600 Peter Morand Cresent, Ottawa, ON K1G 5Z3 Canada; 4 CHU de Québec Research Centre-Université Laval site Hôpital St-Francois d’Assise, 10 Rue Espinay, Quebec City, Quebec, G1L 3L5 Canada; 5 School of Rehabilitation Therapy, Faculty of Health Sciences, Queen’s University, 31 George Street Kingston, Ottawa, ON K7L 3N6 Canada; 6 Children’s Hospital of Eastern Ontario, 401 Smyth Road, Ottawa, ON K1H 8L1 Canada; 7 Learning Services, The Ottawa Hospital, 1053 Carling Ave, Ottawa, ON K1Y 4E9 Canada

**Keywords:** Implementation, Pediatrics, Shared decision-making, Barriers, Facilitators, Systematic review, Taxonomy, Ottawa Model of Research Use

## Abstract

**Background:**

Shared decision-making (SDM) is rarely implemented in pediatric practice. Pediatric health decision-making differs from that of adult practice. Yet, little is known about the factors that influence the implementation of pediatric shared decision-making (SDM). We synthesized pediatric SDM barriers and facilitators from the perspectives of healthcare providers (HCP), parents, children, and observers (i.e., persons who evaluated the SDM process, but were not directly involved).

**Methods:**

We conducted a systematic review guided by the Ottawa Model of Research Use (OMRU). We searched MEDLINE, EMBASE, Cochrane Library, CINAHL, PubMed, and PsycINFO (inception to March 2017) and included studies that reported clinical pediatric SDM barriers and/or facilitators from the perspective of HCPs, parents, children, and/or observers. We considered all or no comparison groups and included all study designs reporting original data. Content analysis was used to synthesize barriers and facilitators and categorized them according to the OMRU levels (i.e., decision, innovation, adopters, relational, and environment) and participant types (i.e., HCP, parents, children, and observers). We used the Mixed Methods Appraisal Tool to appraise study quality.

**Results:**

Of 20,008 identified citations, 79 were included. At each OMRU level, the most frequent barriers were features of the options (decision), poor quality information (innovation), parent/child emotional state (adopter), power relations (relational), and insufficient time (environment). The most frequent facilitators were low stake decisions (decision), good quality information (innovation), agreement with SDM (adopter), trust and respect (relational), and SDM tools/resources (environment). Across participant types, the most frequent barriers were insufficient time (HCPs), features of the options (parents), power imbalances (children), and HCP skill for SDM (observers). The most frequent facilitators were good quality information (HCP) and agreement with SDM (parents and children). There was no consistent facilitator category for observers. Overall, study quality was moderate with quantitative studies having the highest ratings and mixed-method studies having the lowest ratings.

**Conclusions:**

Numerous diverse and interrelated factors influence SDM use in pediatric clinical practice. Our findings can be used to identify potential pediatric SDM barriers and facilitators, guide context-specific barrier and facilitator assessments, and inform interventions for implementing SDM in pediatric practice.

**Trial Registration:**

PROSPERO CRD42015020527

**Electronic supplementary material:**

The online version of this article (10.1186/s13012-018-0851-5) contains supplementary material, which is available to authorized users.

## Introduction

Shared decision-making (SDM) is an evidenced-based approach that promotes collaboration between patients, family members, and healthcare providers (HCP) when making health decisions. By exchanging information about the evidence (options, risks, and benefits) and the patient and family’s preferences and values, HCPs, patients, and family members can deliberate to determine the best treatment plan [1]. This approach to decision-making is considered essential for patient-centered care, has garnered increasing international support among policy makers, and is recommended by pediatric regulatory organizations [2–4]. Nonetheless, implementation of SDM in pediatric healthcare remains limited [5, 6].

Determining the barriers and facilitators that influence the clinical use of evidence-based practices are critical for promoting their uptake [7]. Two systematic reviews have examined the barriers and facilitators of implementing SDM in adult medicine from the perspectives of HCPs and patients [8, 9]. Findings showed that HCPs most commonly perceived time constraints, lack of applicability due to patient characteristics, and lack of applicability due to the clinical situation, as the main barriers [8]. Adult patients perceived power imbalances in the doctor-patient relationship and inadequate knowledge as primary barriers to SDM [9].

Several factors make health decision-making in pediatrics different from adult clinical practice. Children’s evolving developmental context (e.g., biological, cognitive, and psychosocial variables) impacts their participation in health decisions. As such, determining the extent that children should be involved is difficult [10]. Pediatric decision-making is also complicated by the inclusion of multiple stakeholders (i.e., child, family members, and HCPs), each with their own preferences and values [11]. Parents or guardians act as surrogate decision makers. When faced with making difficult decisions on their child’s behalf, parents or guardians must decide without knowledge of “what would the child do or want?”. Further, the legislation and policy about pediatric health decisions can be complex, with different guiding principles depending on state/provincial laws, treatments being considered, and organizational policy [4]. Given this unique context, the barriers and facilitators that influence SDM in pediatrics likely differ from those identified in the adult literature.

Effective implementation of healthcare innovations requires knowledge about the barriers and facilitators influencing its use. When implementation interventions are designed to overcome identified barriers, there is an increased use of the innovation (e.g., SDM) in clinical practice [7]. Barriers and facilitators to knowledge use are also strong predictors of intention and behavior change [12]. In the adult literature, high-quality evidence underpins several implementation interventions, such as patient decision aids, decision coaching, and education and training, which facilitate SDM in clinical consultations [13–15]. Compared to the adult literature, few pediatric SDM implementation interventions have been developed, monitored, or evaluated [16, 17]. A systematic review that evaluated the efficacy of SDM interventions in pediatrics found that of the 54 unique SDM interventions identified, 63% targeted the parents. Only half of these interventions were evaluated. Meta-analysis suggested that SDM interventions might reduce parents’ decisional conflict and improve their knowledge, but the impact on other adopters (e.g., children) was inconclusive [5]. Knowledge about the factors influencing SDM could inform and advance SDM implementation in pediatric practice. Therefore, we identified and synthesized the barriers and facilitators of SDM in pediatric practice from the perspectives of HCPs, parents, children, and observers (i.e., individuals who evaluated SDM, but did not participate in it).

## Methods

### Design

We conducted a systematic review, guided by the Cochrane Handbook for Systematic Reviews [18], and followed the PRISMA reporting guidelines [19]. Our protocol is registered in PROSPERO (ID: CRD42015020527) [20].

### Conceptual model

**Fig. 1:**
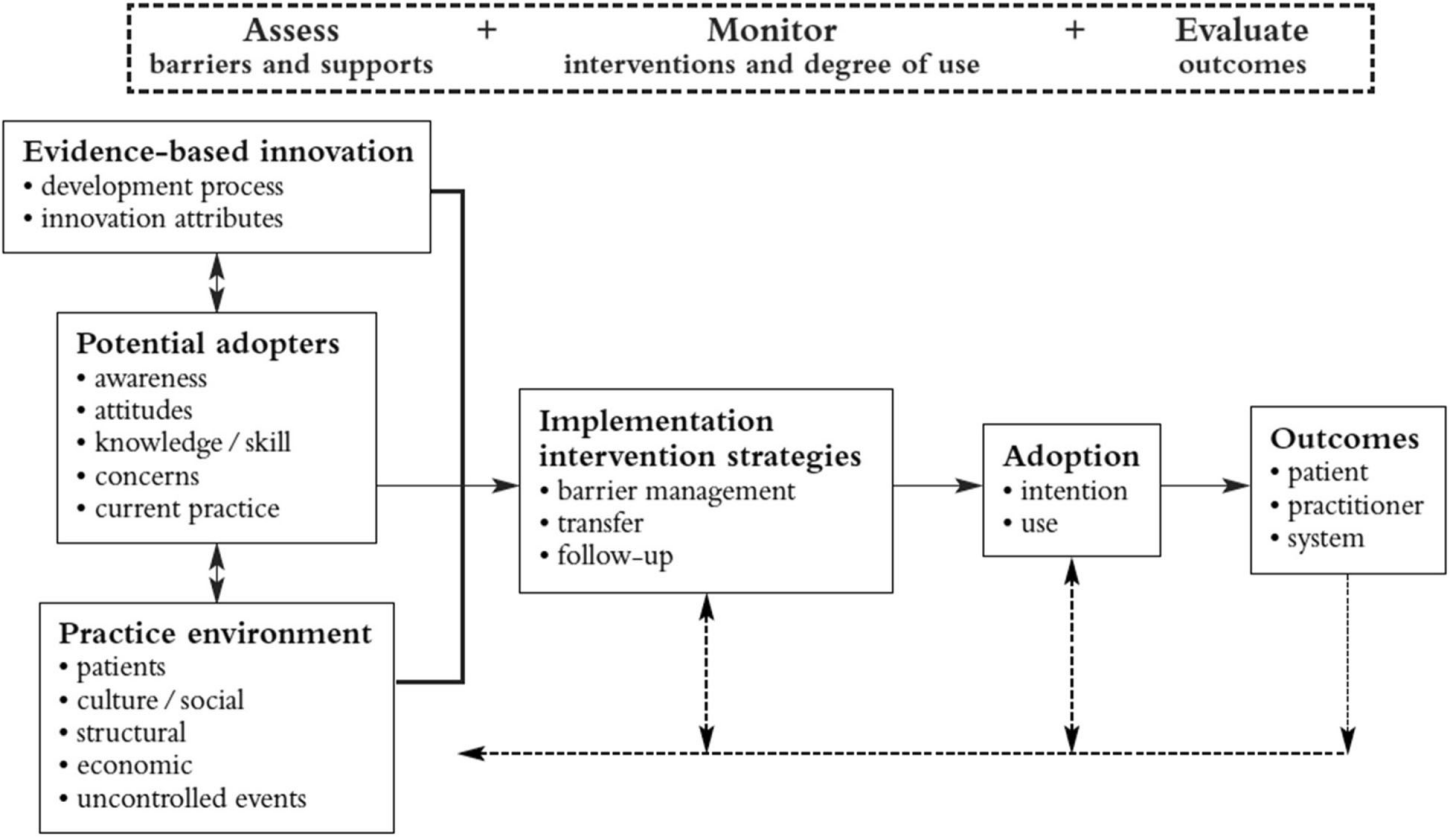
The Ottawa Model of Research Use. Printed with permission from Ian D. Graham

We used the Ottawa Model of Research Use (OMRU) as our guiding theoretical model [21] (Fig. 1). The OMRU is a conceptual model of health research use derived from planned action theories, research utilization, and physician behavior change literature. The model seeks to explain the implementation of evidence into clinical practice using six key components: the innovation (evidence to be implemented), potential adopters, practice environment, implementation interventions, adoption, and outcomes. Given that these components are context-dependent, the OMRU outlines the following iterative process phases for implementing evidence: (1) assess barriers and facilitators related to the innovation, adopters, and practice environment; (2) design and implement interventions to minimize barriers and leverage facilitators; (3) monitor the use of evidence in clinical practice and the implementation process; and (4) evaluate outcomes and impact. Our review focuses on the first process phase of assessing barriers and facilitators.

A primary assumption underpinning the OMRU is that barrier and facilitator assessments are essential for informing the selection of implementation strategies. During the iterative coding and content analysis phase, we added two additional levels to reflect our data: the decision level and the relational level [9, 22]. We defined each OMRU level as follows: (A) the decision level includes influencing factors related to the decision itself or that are antecedent to the SDM process (e.g., features of the options and high or low stake decisions); (B) the innovation was SDM or a collaborative decision-making approach between HCPs, parents, and/or children; (C) adopters are the individuals who use the innovation, in this case, HCPs, parents, and children; (D) relational represents the interpersonal interactions and processes between the HCP, patient, and family during the SDM discussions [22]; and, (E) the practice environment, in this case the pediatric clinical setting, which includes structural factors (e.g., legislation, policy, physical structures, and workload).

### Inclusion criteria

We used the PICOS framework to guide our eligibility criteria [23] (Table 1). Eligible participants included HCPs (e.g., frontline staff of any discipline, clinical managers, and administrators), parents or guardians (collectively referred to as parents), children aged 18 years or less, and observers. Observers are individuals who were not involved in the pediatric SDM process, but evaluated SDM in some way (e.g., research assistants who evaluated SDM in person or in videotaped consultations using a validated instrument). Observers differ from adopters, in that adopters are involved in the SDM process. We collectively refer to HCPs, parents, children, and observers as “participants.” The intervention was SDM or a collaborative decision-making approach regarding a decision about a child’s health [1, 10]. Outcomes were barriers or facilitators of SDM in pediatric clinical practice reported in the results section of the included study. We excluded studies that reported on barriers and facilitators of health decisions for combined pediatric and adult patient populations (e.g., family practice primary care). We included all study designs with original data, with or without comparison groups. There were no language restrictions. These parameters are consistent with previous systematic reviews that examined SDM barriers and facilitators in adult clinical practice [8, 24].

**Table 1:** Study eligibility criteria

	Included	Excluded
Participants	Healthcare providers Parents, guardians, and/or caregivers Children 18 years of age or younger Observers	Adult patients (19 years and older) and individuals involved in making a decision about the health of an adult patient
Intervention	SDM in the pediatric clinical context A collaborative decision-making approach consistent with SDM	Non-SDM interventions Hypothetical decisions Health decisions in a non-clinical setting (e.g., schools) Decisions about pregnancy, perinatal care (before birth) Decisions about participation in research
Comparison	All comparison groups, including none	
Outcomes	Barriers and/or facilitators of SDM in pediatric clinical and/or health care practice Note: outcomes had to be reported in the results section of the paper	All other SDM outcomes (e.g., impact of a SDM intervention)
Study methods	All study designs with original data	Reviews Commentaries Unpublished studies

### Information sources and search strategy

An information specialist (AD) designed the search strategy and conducted electronic searches specific to each database with input from our research team. The search was designed to target SDM barriers and facilitators in pediatric clinical practice (see Additional file 1). We searched the following electronic databases (from inception to March 2017): MEDLINE, EMBASE, Cochrane Library, PubMed, PsycINFO, and CINAHL. Review of reference lists of included studies did not identify additional studies.

### Study selection

We uploaded citations onto a title and abstract screening web application, designed by an information technologist (AS) at our research institute. This application allowed reviewers (LB, KL, JJ, DS, AS) to independently evaluate study eligibility in a three-stage screening process. First, titles were randomly assigned to two independent reviewers and screened to determine their relevance to decision-making in pediatrics. Reviewers did not know if they were screening first or second and indicated whether an article was “included,” “excluded,” or “unsure” based on the eligibility criteria. Both reviewers were required to determine that an article was excluded for it to be screened out, while titles deemed “included” or “unsure” by at least one reviewer moved to the second screening stage. We followed the same process for abstracts. Finally, two reviewers independently read full texts to determine eligibility. At this stage, reviewers reached consensus for study inclusion and exclusion.

### Data collection

Two reviewers independently extracted data using a standardized and pre-piloted data extraction form. We extracted citation information (e.g., country of origin, language), study information (e.g., objectives, design, and methodological approaches), participant types (i.e., HCP, parent, child, and observer), and findings (i.e., barriers and facilitators). Inconsistencies in extracted data were resolved through consensus, as outlined by the Cochrane Handbook for Systematic Reviews [18].

### Analysis

Pooling of quantitative data was inappropriate due to the heterogeneity across included studies regarding design, decision type and timing, adopters involved, methods, and measures used. We synthesized the barriers and facilitators using deductive and inductive content analysis. This involved becoming familiar with the data, identifying units of relevant data, open-coding, category development, compiling data, and iterative data comparison between coders [25, 26]. We transferred the extracted text representing the barriers and facilitators into NVivo qualitative analysis software (NVivo; QSR International Pty Ltd. V10, 2012). Two coders conducted the analysis, which was informed by taxonomies derived from systematic reviews of SDM barriers and facilitators in adult clinical practice, from the perspectives of healthcare providers and patients [8, 9]. These taxonomies describe a range of factors that influence the implementation of SDM in adult clinical practice. Categories were organized under the OMRU levels (i.e., decision, innovation, adopter, relational, and environmental). An advantage of using the OMRU to categorize findings is that interventions can be selected to target the barriers at the level in which the barrier is occurring. Then, we rank-ordered the influential factor according to the frequency of studies that reported it. We counted the barrier and facilitator frequency once per study. Specifically, if one paper reported the same barrier or facilitator multiple times, we counted it once. However, if the same factor was reported as both a barrier and facilitator, we counted it once for each a barrier and facilitator. When a study reported multiple perspectives (e.g., HCPs and parents), and each participant type reported the same barrier or facilitator, we counted the factor once (as defined above) for each participant type.

In summary, our analysis used a complementary approach to promote a theory-driven and evidence-based deduction, induction and categorization of pediatric SDM barriers and facilitators. First, we drew from systematic reviews on SDM barriers and facilitators in adult practice to ensure a comprehensive assessment of SDM barriers and facilitators within our included studies. Second, we used the OMRU to categorize these findings in a manner conducive to informing future pediatric SDM implementation efforts.

### Quality assessment

Two independent raters appraised study quality using the Mixed Method Appraisal Tool (MMAT) [27, 28]. The MMAT criteria (Table 3) were developed based on a thematic analysis of the quality appraisal processes revealed by health-related systematic reviews. The tool was designed to concurrently appraise qualitative, quantitative, and mixed method studies for large and complex systematic reviews [27]. The MMAT reliability is reported to range from fair to perfect [28] and is well suited for the assessment of complex interventions that are context-dependent and process-oriented, such as SDM. We report items scores at the individual study level (Table 3) and overall (see the “Study appraisal” section). Raters resolved discrepancies through discussion and consensus.

## Results

### Identified studies and characteristics

**Fig. 2:**
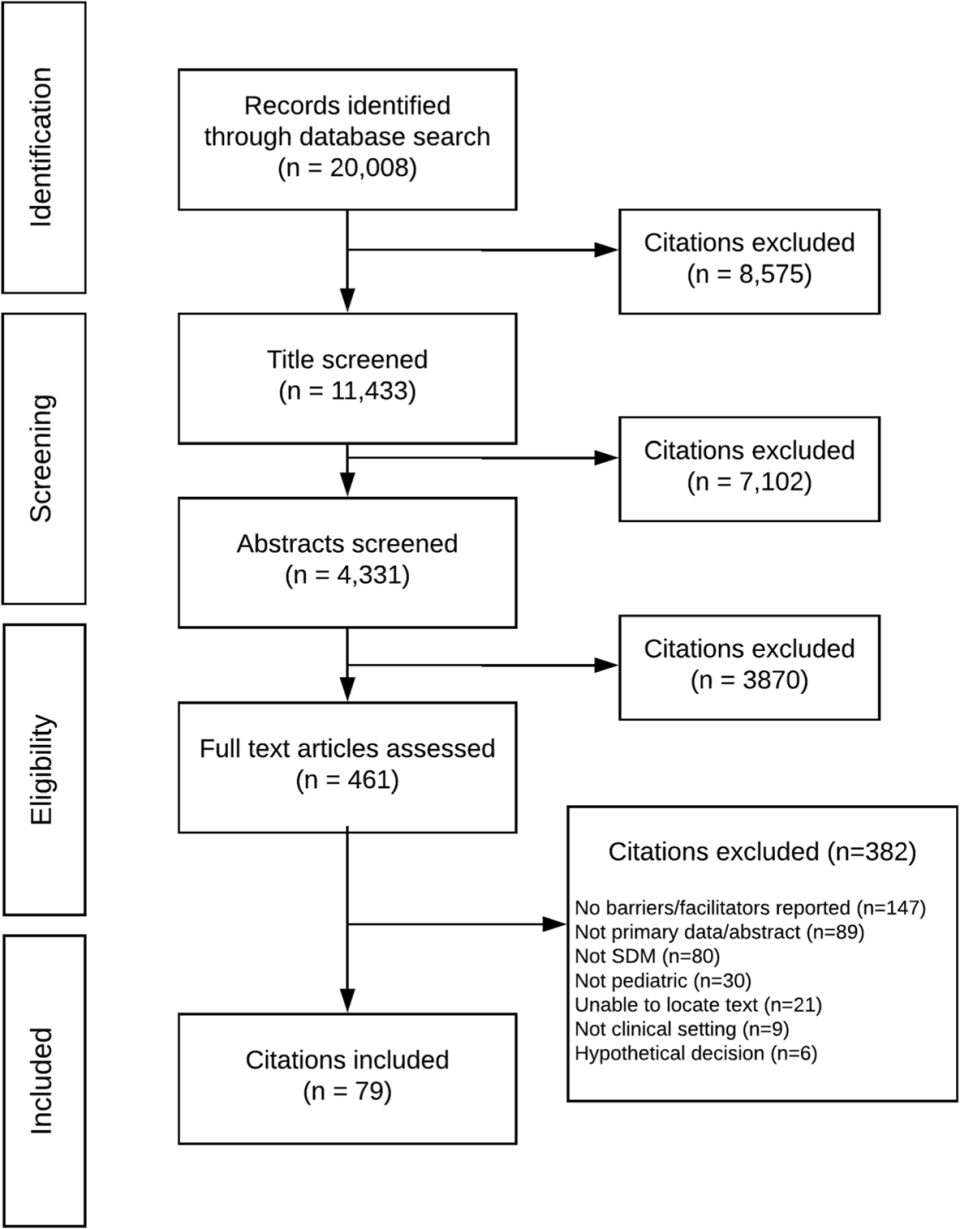
Literature flow chart

**Fig. 3:**
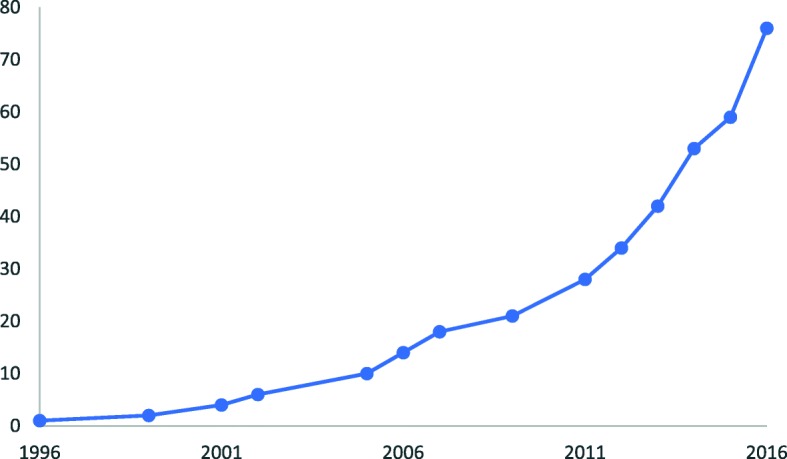
Cumulative citation count (1996–2016)

Our search yielded 20,008 citations (Fig. 2). After removing duplicates and screening titles and abstracts, we examined 461 full-text articles, of which 79 publications (representing 78 distinct studies) were included. Included studies were published between 1996 and 2017, with increasing publications over time (Fig. 3).

All studies were published in English except one, which was French [29]. Studies originated from 15 countries: the USA (*n* = 34), the UK (*n* = 13), Canada (*n* = 9), Ireland (*n* = 5), Sweden (*n* = 3), Australia (*n* = 3), the Netherlands (*n* = 2), one study from each of France, Italy, Israel, Kenya, South Africa, Switzerland, Amsterdam, Turkey, and both Canada and the USA together (Table 2).

**Table 2:** Characteristics of the included studies

Author, year, country of origin	Study objective related to this systematic review	Methodological approach*	Design	Data source*	Response rate*	Participants*
Citations reporting healthcare professional perceptions only						
Abrines-Jaume, 2016, UK [50]	To explore the implementation of SDM in pediatric mental health services and identify clinician-determined facilitators to SDM	Qualitative	Secondary analysis of a larger study	Log-book of post-encounter stories	NR	23 HCPs (psychiatrists, psychologists, nurses, family therapists, social workers, play therapists)
Andre, 2005, France [29]	To describe how pediatric residents involve children during medical decision-making	Quantitative	Prospective descriptive	Survey	75%	43 HCPs (pediatric residents)
Bejarano, 2015, USA [51]	To evaluate the feasibility of implementing SDM practices in pediatrics and to assess physicians’ satisfaction with SDM	Mixed methods	Pre-post study	Survey	NR	5 HCPs (physicians)
Boss, 2009, USA [52]	To determine fellows’ training in communication and decision and their perceived preparedness to lead family discussions	Mixed methods	Descriptive	Survey	72%	101 HCPs (pediatric fellows)
Delany, 2017, Australia [53]	To get clinicians’ views about resources designed to aid parents facing end-of-life decisions for their child	Qualitative	Descriptive	Interviews	NR	18 HCP (not specified)
Dodds, 2016, USA [54]	To understand pediatric physicians’ use SDM and their perceptions of barriers and facilitators to SDM for decisions about tumor necrosis factor-α inhibitor treatment	Quantitative	Descriptive	Survey	66%	196 HCPs (physicians)
Fay, 2016, USA [55]	To assess the impact, acceptability, and feasibility of a tool designed to enhance SDM	Mixed methods	Descriptive	Interview	80%	4 HCPs (physicians)
Frize, 2013, Canada [56]	To translate information, using a decision support tool, for parents making decisions in the neonatal intensive care unit.	Mixed methods	Descriptive	Not specified	NR	5 HCPs (physicians)
Honeycutt, 2005, USA [57]	To examine physician reported use of participatory decision-making with children/parents	Quantitative	Descriptive	Survey	47%	219 HCPs (physicians)
Lee, 2006, USA [58]	To explore clinician views and practices regarding assent and compare practice with existing guidelines	Mixed methods	Descriptive	Interviews and questionnaire	NR	35 HCPs (physicians, nurses, physician assistants)
Lipstein, 2013, USA [59]	To understand the barriers and facilitators to SDM for juvenile idiopathic arthritis	Qualitative	Descriptive	Interviews	NR	10 HCPs (physicians and nurses)
Miller, 2001, UK [60]	To investigate the ways children’s nurses see themselves facilitating youth in decision-making	Qualitative	Descriptive	Interviews and focus groups	NR	8 HCPs (nurses)
Partridge, 2005, South Africa [61]	To characterize South African pediatricians’ practices and attitudes related to parent counseling and life-support decisions for premature infants	Quantitative	Descriptive	Survey	24%	394 HCPs (physicians)
Runeson, 2001, Sweden [62]	To identify factors influencing children’s participation in healthcare decision-making	Qualitative	Critical incident technique	Open-ended questionnaire	40%	140 HCPs (physicians, nurses, assistant nurses, play therapists, psychologists)
Schalkers, 2016, Amsterdam [63]	To investigate HCPs’ perspectives on, improving, child participation, in pediatric hospital care	Qualitative	Descriptive	Interviews	NR	10 HCPs (heads of wards, physicians, nurses, play therapists, manager, communication advisor, policy advisor)
Simmons, 2013, Australia [64]	To explore clinicians’ experiences and beliefs about treatment decision-making for youth diagnosed with depressive disorders	Qualitative	Descriptive	Interviews	NR	22 HCPs (psychologists, psychiatrists, physicians, nurses, youth workers)
Shirley, 2015, USA [65]	To describe the production, implementation, and evaluation of a decision aid for pediatric orthopedics	Quantitative	Descriptive	Questionnaire	NR	4 HCPs (physicians)
Tam-Seto, 2015, Canada [66]	To better understand SDM in adolescent mental health using the Canadian Model of Client-Centered Enablement	Qualitative	Critical incident technique	Interviews	NR	6 HCPs (occupational therapists)
Vaknin, 2011, Isreal [67]	To examine HCPs’ attitudes, perceptions, and reported practices regarding the inclusion of pediatric patients in simple decisions	Quantitative	Descriptive	Survey	66%	143 HCP (nurses and physicians)
Citations reporting child perceptions only						
Coyne, 2012, Ireland [68]	To elicit children’s perspectives on participation in information sharing and decision-making	Qualitative	Descriptive	Interviews	NR	55 children (aged 7–18)
Coyne, 2011, Ireland [69]	To explore hospitalized children’s experiences and preferences for participation in decision-making	Qualitative	Descriptive	Interviews and focus groups	82%	55 children (aged 7–18)
Kelly, 2016, USA [70]	To better understand how children viewed their treatment decision-making involvement	Qualitative	Descriptive	Interviews	NR	29 children (aged 9–17)
Kelsey, 2007, UK [71]	To explore children’s perceptions of their involvement in healthcare decisions	Qualitative	Descriptive	Audio diary and interview	NR	10 children (aged 12–16)
Koller, 2017, Canada [72]	To examine how children with chronic medical conditions view healthcare education and decision-making	Qualitative	Descriptive	Interviews	NR	26 children (aged 5–18)
Lambert, 2013, Ireland [73]	To describe information exchange between HCPs and children in the hospital	Qualitative	Descriptive	Interviews and observation	NR	49 children (aged 6–16)
Lipstein, 2013b, USA [74]	To understand adolescents’ roles and preferences in chronic disease treatment decisions	Qualitative	Descriptive	Interviews	75%	15 children (aged 11–18)
Weaver, 2015, USA [75]	To investigate decision-making preferences of child oncology patients and parent/clinician behaviors that support their preferred decision-making role	Qualitative	Descriptive	Interviews	78%	40 children (aged 12–18)
Citations reporting parent perceptions only						
Butler, 2014, USA [76]	To investigate perceptions of SDM among low-income minority parents of children referred to mental health services	Quantitative	Descriptive	Questionnaire	69%	36 parents (from minority groups)
Butler, 2015a, USA [77]	To examine associations between parental reported SDM and parental perceptions of children’s mental health care	Quantitative	Descriptive	Survey	NR	21,721 parents
Butler, 2015b, USA [78]	To examine whether SDM varies by child health and whether receiving medical home care attenuates differences in SDM among child health conditions.	Quantitative	Descriptive	Survey	NR	21,721 parents
Fiks, 2010, USA [79]	To identify SDM patterns among children with attention-deficit/hyperactivity disorder or asthma and determine if demographics, health status, or access to care are associated with SDM	Quantitative	Cross-sectional descriptive	Survey database	NR	4135 parents (person from the household who knew the most about the child’s health)
Gkiousias, 2016, UK [80]	To explore parents’ decision-making process for pediatric management otitis media with effusion	Qualitative	Descriptive; subgroup analysis of larger study	Interviews	NR	12 parents
Hummelinck, 2007, UK [81]	Explore parents’ perspectives on the relationship with HCPs and their involvement in decisions about their child’s care	Qualitative	Descriptive	Interviews	51%	23 parents
Kline, 2012, USA [82]	To evaluate family satisfaction and decision-making with a pediatric hematology–oncology palliative care program	Mixed methods	Descriptive	Interviews and survey	56%	20 parents (or guardians)
Lerret, 2016, USA [83]	To report parents’ medical decision-making experiences for children who had a solid organ transplant	Qualitative	Prospective, longitudinal	Interviews	86%	48 parents
Li, 2016, Canada [84]	To explore parents’ perceptions of decisional needs for genome-wide sequencing for their child	Qualitative	Interpretive descriptive	Interviews	71%	15 parents
Mack, 2011, USA [85]	To evaluate parents’ involvement and preferences for decision-making regarding their child’s cancer care	Quantitative	Descriptive	Survey	70%	194 parents
Mak, 2014, Canada [86]	To understand parents’ perspectives on decision-making for child anxiety treatment and to identify ways to promote parental involvement in treatment decisions	Qualitative	Descriptive	Interviews	68%	19 parents
Pyke-Grimm, 2006, USA [87]	To determine factors that parents identified as influencing their role in treatment decision-making for their child with cancer	Mixed methods	Descriptive	Interviews and questionnaires	NR	36 parents
Rosati, 2017, Italy [88]	To explore general parental views on SDM and patient-physician SDM relationships in pediatric outpatients’ clinic	Quantitative	Descriptive	Survey	85%	458 parents (one grandmother)
Smalley, 2014, USA [89]	To determine families’ perceptions of SDM in their child’s health care and correlates of perceived SDM	Quantitative	Cross-sectional descriptive	Survey database	NR	11,102 parents (weighted)
Valenzuela, 2014, USA [90]	To describe caregiver-report of SDM with their child’s health care provider with youth with type 1 diabetes	Quantitative	Descriptive	Database	NR	439 parents (described as caregivers of the child)
Walker-Vischer, 2015, USA [91]	To describe the experience of Latino parents of hospitalized children during family-centered rounds	Qualitative	Descriptive	Survey	85%	17 parents (minority group)
Walter, 2016, USA [92]	To learn about parent’s experiences of having goals of care discussions with their child’s HCP	Mixed methods	Descriptive	Interviews and survey	75% (survey)	55 parents (one other)
Xu, 2004, USA [93]	To explore whether there are ethnic differences in parents’ perceptions of the participatory styles of their children’s physicians	Quantitative	Descriptive	Survey	52%	5941 parents (described as households)
Yin, 2012, USA [94]	To assess whether parental health literacy is associated with differences in perceived barriers to care and attitudes regarding participatory decision-making with the HCP	Quantitative	Descriptive	Questionnaire	71%	823 parents (or legal guardian)
Citations reporting observers’ perspectives only						
Brinkman, 2011, USA [95]	To describe physician behavior during treatment-planning encounters for children newly diagnosed as having ADHD	Quantitative	Prospective cohort	Video-recorded clinical consultations	65% for parents	26 observed consultations
Cahill, 2007, UK [96]	To identify interaction features between doctors, children, and their caregivers in the consultation that are associated with the child’s participation	Qualitative	Descriptive	Video-recorded clinical consultations	8% of HCPs	31 observed consultations
Elwyn, 1999, UK [97]	To examine the feasibility of SDM in consultations, when conflict occurs between parents and clinicians, about antibiotics for an upper respiratory tract infection	Qualitative	Descriptive	Recordings of consultation discourse	NR	2 observed consultations
Hallstrom, 2002, Sweden [98]	To investigate the extent to which parents participate in decisions concerning their hospitalized child’s care and identify factors influencing a parent’s participation	Quantitative	Observational	Field notes of observations	96%	35 parents of 24 children were observed
Lipstein, 2014, USA [99]	To understand how decisions about higher-risk treatments are made for pediatric chronic conditions	Qualitative	Descriptive	Video-recorded clinical consultations	91% of HCPs	21 observed consultations
Runeson, 2002, Sweden [100]	To illustrate children’s participation in decision-making and various levels of participation	Mixed methods	Observational	Ratings and field notes of observations	96%	135 observed hours of 24 hospitalized children
Wiering, 2016, the Netherlands [101]	To explore how oncologists involve families in SDM and which factors are associated with this process	Quantitative	Descriptive	Rating scale	NR	43 observed consultations
Citations reporting multiple perspectives						
Angst, 1996, USA [102]	To describe how children with chronic illness and their parents are involved in health care decisions	Qualitative	Secondary analysis	Interviews	NR	16 parents; 28 children
Astbury, 2017, UK [103]	To explore the processes that support SDM when HCPs and parents are creating plans to improve the well-being of children	Qualitative	Descriptive	Interviews	NR	11 HCPs (not specified); 11 parents
Beck, 2014, Canada [104]	To examine the treatment decision-making process for children hospitalized with newly diagnosed immune thrombocytopenia	Qualitative	Descriptive	Focus groups	NR	10 HCP; 16 parents; 7 children
Boland, 2016, Canada [105]	To explore barriers and facilitators to implementing SDM and decision support in a children’s hospital	Qualitative	Interpretive descriptive	Interviews and focus groups	Convenience sample	35 HCP; 15 parents; 7 children
Coyne, 2006, Ireland [106]	To explore children’s, parents’, and nurses’ views on participation in care in the healthcare setting	Qualitative	Grounded theory	Interviews, observations, and drawings	NR	12 HCPs; 10 parents; 11 children
Coyne, 2014, Ireland [107]	To explore children with cancer's participation in SDM and identify confounding and facilitating factors that influence children’s participation in SDM	Qualitative	Descriptive	interviews	NR	40 HCP; 22 parents; 20 children
Daboval, 2016, Canada [108]	To document interactions between parents and neonatologists that parents linked to their satisfaction with SDM	Qualitative	Multiple-case ethnomethodological study	Interviews	NR	6 HCPs; 10 parents
Fiks, 2011, USA [109]	To compare how parents and clinicians understand SDM in attention-deficit/hyperactivity disorder	Qualitative	Descriptive	Interviews	100% HCPs; NR parents	30 HCPs; 60 parents
Garnett, 2016, UK [110]	To explore child-parent SDM for childhood asthma management	Qualitative	Descriptive	Interviews	NR	9 parents; 8 children
Heath, 2016, UK [111]	To explore how parents and HCPs make decisions regarding putting children forward for pediatric epilepsy surgery	Qualitative	Descriptive, observational	Interviews	NR	10 HCPs; 9 parents
Iachini, 2015, US [112]	To explore youth and parent perspectives of practitioner behaviors important for fostering treatment engagement	Qualitative	Exploratory	Focus group	NR	11 parents; 19 children
Kahveci, 2014, Turkey [113]	To examine SDM in management of critically ill children and the experiences of parents, physicians and nurses	Qualitative	Descriptive	Interviews	72% physicians; 69% nurses; 28% parent	17 HCPs; 6 parents
Karnieli-Miller, 2009, Israel [114]	To analyze SDM regarding medical treatment in real-time encounters	Qualitative	Phenomenological	Interviews and observations	NR	17 HCPs; 17 observed consultations
Kavanaugh, 2005, USA [115]	To describe life support decision-making and the decision support needs of parents, physicians, and nurses for extremely premature infants	Qualitative	Collective case study	Interviews	NR	8 HCPs; 6 parents
Lecouturier, 2015, UK [116]	To explore management and treatment of intermittent distance exotropia decisions and what can be done to support decision-making for clinicians, parents and children	Qualitative descriptive	Descriptive	Interviews	NR	21 HCPs; 37 parents
Levy, 2016, USA [117]	To describe influences on SDM between primary care pediatricians and parents of children with autism	Qualitative	Descriptive	Interviews	22% for HCPs; NR parents	20 HCPs; 20 parents
Markworo, 2014, Kenya [118]	To determine parental involvement in decision-making about their hospitalized children	Mixed methods	Descriptive cross sectional	Interviews and questionnaires	83% HCPs; 88% parents	144 HCPs; 161 parents
Miller, 2009, USA [119]	To explore parent–child collaborative decision-making for chronic illness management	Qualitative	Descriptive	Interviews and focus groups	NR	16 parents; 18 children
Pentz, 2012, Canada and USA [120]	To create a theory of family decision-making regarding pediatric allogeneic transplantation for the treatment of childhood cancer	Mixed methods	Descriptive	Interviews	NR	192 parents 5 children
Ruhe, 2016, Switzerland [121]	To explore how patient participation was put into practice in a pediatric oncology setting	Qualitative	Secondary analysis from larger study; descriptive	Interviews	NR for HCPs; 81% for children; 90% for parents	16 HCPs; 19 parents; 17 children
Sajeev, 2016, Australia [122]	To develop and pilot test a decision aid to assist parents making cancer or a hematological decisions with their HCPs	Mixed methods	Observational pilot	Questionnaires/open-ended questions	65% for HCPs; 72% for parents;	15 HCPs; 31 parents
Sleath, 2011, USA [123]	To examine the extent HCPs engaged in SDM with caregivers and children and factors associated with question asking and SDM	Quantitative	Cross sectional	Interviews and observations	95% HCP 88% parents (some children in sample, not separated)	41 HCPs; 320 parents (some children in sample, not separated)
Smith, 2013, UK [124]	To investigate parent–HCP SDM during the diagnosis of suspected shunt malfunction and their perceptions/experiences of SDM within this clinical context	Mixed methods	Descriptive	Interviews, questionnaires, and video-taped interactions	NR	14 HCPs; 28 parents
Stille, 2013, USA [125]	To describe factors that influence parent–clinician partnerships in SDM when children are referred to subspecialists	Qualitative	Descriptive	Focus groups	NR	23 HCPs; 19 parents
Young, 2006, UK [126]	To explore SDM in the context of community-based physiotherapy services for children with cerebral palsy	Qualitative	Descriptive	Interviews and focus group	NR	10 HCPs; 10 parents; 11 children
Zwaanswijk, 2007, the Netherlands [127]	To explore interpersonal, informational, and decisional preferences of participants involved in pediatric oncology	Qualitative	Descriptive	Online focus groups	29% parents; 36% children; 34% adults with childhood experience	18 parents; 27 children + 32 adults with childhood illness experience

*Reflects those that are pertinent to the research question of this study

Barriers and facilitators were reported from the perspective of HCPs (*n* = 19), parents (*n* = 18), children (*n* = 8), multiple perspectives (*n* = 26), and observers (*n* = 7). Data from 47,363 participants were synthesized, including 45,094 parents (95%), 1785 HCPs (4%), and 484 children (1%). We also included data from more than 138 observed consultations (*n* = 6) plus 135 observed consultation hours (*n* = 1). Observer studies primarily reported on the HCP’s behavior.

### Study appraisal

The MMAT appraisal results are shown in Table 3. Included studies used qualitative (*n* = 47; 60%), quantitative (*n* = 18; 23%), and mixed methods (*n* = 14; 18%). For qualitative studies, 100% of studies reported sources of data relevant to address the research question. Sources of bias included not reporting how: findings related to researchers’ influence (*n* = 39/47, 83% missed), findings related to context (*n* = 12/47, 26% missed/unsure), and the process for analysis was relevant to address the research question (*n* = 4/47, 9% missed/unsure).

**Table 3:** Results of the MMAT appraisal

MMAT items	Qualitative studies	Are the sources of data relevant to address the research question?	Is the process for analyzing data relevant to address the research question?	Is appropriate consideration given to how findings relate to the context?	Is appropriate consideration given to how findings relate to researchers’ influence?	Quantitative descriptive studies	Is the sampling strategy relevant to address the research question?	Is the sample representative of the population under study?	Are the measurements appropriate?	Is there an acceptable response rate (60% or above)?	Mixed methods	Is the research design relevant to address the qualitative and quantitative research questions?	Is the integration of qualitative and quantitative data relevant to address the research questions?	Is appropriate consideration given to the limitations associated with this integration?
Qualitative studies														
Abrines-Jaume, 2016 [50]		○	○	○	○									
Angst, 1996 [102]		○	○	●	●									
Astbury, 2017 [103]		○	○	○	●									
Beck, 2014 [104]		○	○	○	●									
Boland, 2016 [105]		○	○	○	●									
Cahill, 2007 [96]		○	○	○	○									
Coyne, 2006 [106]		○	○	●	●									
Coyne, 2011 [69]		○	○	○	●									
Coyne, 2012, [68]		○	○	○	●									
Coyne, 2014 [107]		○	○	○	●									
Daboval, 2016 [108]		○	○	●	●									
Delany, 2017 [53]		○	○	○	●									
Elwyn, 1999 [97]		○	○	●	●									
Fiks, 2011 [109]		○	○	○	●									
Garnett, 2016 [110]		○	○	○	●									
Gkiousias, 2016 [80]		○	○	○	●									
Hallstrom, 2002 [98]		○	○	○	●									
Heath, 2016 [111]		○	○	○	●									
Hummelinck, 2007 [81]		○	○	●	●									
Iachini, 2015 [112]		○	○	○	●									
Karnieli-Miller, 2009 [114]		○	○	**ο**	●									
Kavanaugh, 2005 [115]		○	**ο**	●	●									
Kahveci, 2014 [113]		○	●	○	●									
Kelly, 2016 [70]		○	○	○	○									
Kelsey, 2007 [71]		○	○	○	●									
Koller, 2017 [72]		○	○	○	○									
Lambert, 2013 [73]		○	○	●	●									
Lecouturier, 2015 [116]		○	○	●	●									
Lerret, 2016 [83]		○	○	○	●									
Levy, 2016 [117]		○	○	○	●									
Li, 2016 [84]		○	○	○	●									
Lipstein, 2013 [59]		○	○	○	●									
Lipstein, 2013b [74]		○	○	○	●									
Lipstein, 2014 [99]		○	○	○	○									
Mak, 2014 [85]		○	○	○	●									
Miller, 2001 [60]		○	**ο**	●	●									
Miller, 2009 [119]		○	○	●	●									
Ruhe, 2016 [121]		○	○	○	●									
Runeson, 2001 [62]		○	○	●	●									
Schalkers, 2016 [65]		○	○	○	●									
Simmons, 2013 [64]		○	○	○	○									
Stille, 2013 [125]		○	○	○	○									
Tam-Seto, 2015 [66]		○	○	○	●									
Walker-Vischer, 2015 [91]		○	○	○	●									
Weaver, 2015 [75]		○	○	○	○									
Young, 2006 [126]		○	○	○	●									
Zwaanswijk, 2007 [127]		○	**ο**	○	●									
Quantitative Studies														
Andre, 2005 [29]							○	○	●	○				
Brinkman, 2011 [95]							○	●	○	○				
Butler, 2014 [76]							○	○	○	○				
Butler, 2015; 2015b [77, 78]							○	○	○	**ο**				
Dodds, 2016 [54]							○	**ο**	○	○				
Fiks, 2010 [79]							○	○	○	○				
Honeycutt, 2005 [57]							○	○	○	●				
Mack, 2011 [85]							○	○	○	○				
Partridge, 2005 [61]							○	○	●	●				
Rosati, 2017 [88]							○	○	●	○				
Smalley, 2014 [89]							○	○	○	○				
Shirley, 2015 [65]							○	●	○	○				
Vaknin, 2011 [67]							○	○	○	○				
Valenzuela, 2014 [90]							○	○	○	○				
Wiering, 2016 [101]							○	○	○	○				
Xu, 2004 [93]							○	**ο**	**ο**	●				
Yin, 2012 [94]							○	**ο**	○	○				
Mixed methods studies														
Bejarano, 2015 [51]		○	○	●	●		○	●	○	○		○	○	○
Boss, 2009 [52]		○	○	●	●		○	○	○	○		○	**ο**	●
Fay, 2016 [55]		○	○	●	●		○	○	○	○		○	○	●
Frize, 2013 [56]		○	**ο**	●	●		○	●	**ο**	**ο**		○	**ο**	**ο**
Kline, 2012 [82]		○	○	●	●		○	**ο**	○	●		○	○	●
Lee, 2006 [58]		○	○	○	●		○	●	○	**ο**		○	**ο**	●
Markworo, 2014 [118]		○	○	●	●		○	○	●	○		○	○	●
Pentz, 2012 [120]		○	○	○	●		○	○	**ο**	○		○	○	●
Pyke-Grimm, 2006 [87]		○	○	○	●		○	●	○	○		○	○	●
Runeson, 2002 [100]		○	○	●	●		○	○	●	**ο**		○	○	●
Sajeev, 2016 [122]		○	○	○	●		○	○	○	○		○	○	●
Sleath, 2011 [123]		○	○	○	●		○	○	○	○		○	○	●
Smith, 2013 [124]		○	○	●	●		**ο**	**ο**	○	**ο**		○	○	●
Walter, 2016 [92]		○	○	●	●		○	○	●	○		○	○	●

*○ = yes; ● = no; **ο** = unsure

For quantitative studies, all studies received credit for having a sampling strategy relevant to the research question. Sources of bias included not reporting if the sample was representative (*n* = 5/18, 28% missed/unsure), if the measures used were appropriate (i.e., of known origin, valid, or standardized) (*n* = 4/18; 22% missed/unsure), and whether the response rate was 60% or above (*n* = 4/18, 22% missed/unsure).

The mixed method studies had the lowest ratings. All studies received credit for having relevant sources of data appropriate for the research question and a research design that was relevant to address a qualitative and quantitative research question. Other sources of bias were how findings related to researchers’ influence (*n* = 14/14, 100% missed), how limitations associated with integration of qualitative and quantitative approaches (*n* = 13/14, 93% missed), how findings related to the context (*n* = 9/14, 64% missed), if the sample was representative of the population under study (*n* = 6/14, 42% missed), if measurements used were appropriate (*n* = 5/14, 36% missed), if an acceptable response rate was reported (*n* = 5/14, 36% missed), integration of qualitative and quantitative data relevant to address the research question(s) (*n* = 3/14, 21% missed), if the data analysis was relevant to the research question (*n* = 1/14, 7% unsure), and if the sampling strategy was relevant to address the research question (*n* = 1/14, 7% unsure).

### Pediatric SDM barriers and facilitators

We report our findings in several formats, including a narrative report of frequently cited barriers and facilitators under each OMRU level (below), a detailed taxonomy of pediatric SDM barriers and facilitators, including frequency counts across OMRU levels and participant types (Table 4), and influential factors (not separated into barriers and facilitators) mapped to the OMRU (Fig. 3).

**Table 4:** Taxonomy and frequency counts of pediatric SDM barriers and facilitators from multiple perspectives

Influencing factor (# unique studies)	Citations	Barrier (B) and facilitator (F) (frequency counts)									
		HCP		Parent		Children		Observer		Total	
Decision level (19)		B	F	B	F	B	F	B	F	B	F
Option features (11)	[67, 70, 84, 99, 109, 116, 117, 119, 120, 124, 125]	4		7		3	2		1	14	3
High versus low stake decisions (9)	[43, 46, 49, 50, 52, 84, 87, 99, 101]	2	3	1	2	1	4			4	9
Availability of medical and research information (8)	[67, 70, 84, 99, 107, 117, 119, 120, 124, 125]			4		1	2			5	2
Atypical decision or uncomfortable topics (2)	[64, 119]	1	1				1			1	1
Totals		7	3	12	2	5	9		1	24	15
Innovation level (i.e., SDM) (34)		B	F	B	F	B	F	B	F	B	F
Level of quality/tailored information that is given to the family (30)	[54, 59, 60, 64, 68–70, 72, 73, 75, 80, 82–84, 91, 92, 96, 97, 102, 104, 106, 108, 109, 111, 112, 115, 116, 121, 124, 125]		8	4	11	8	9	1	1	13	31
Impact of SDM on time (7)	[50, 56, 64, 65, 104, 105, 116]	5	2				1			5	3
Totals		5	10	4	11	8	10	1	1	18	34
Adopter level (i.e., HCP, parent, and child) (70)		B	F	B	F	B	F	B	F	B	F
Attitudes (43)											
Agree with/desire for SDM/DM involvement (31)	[58, 60, 64, 67–70, 72–75, 80, 81, 83, 85–88, 94, 102, 106, 109, 112, 113, 116, 119, 121, 124, 125, 127]	1	5	2	15	7	11			10	31
Beliefs about consequences (7)	[29, 30, 32, 50, 85, 99, 93, 95, 104]		4		3	1	3			1	10
Parents/children cannot understand information (6)	[56, 64, 70, 91, 105, 115]	5		1						6	
Beliefs about capabilities (6)	[59, 61, 64, 81, 103, 113, 124]	3	2	2	1	1				6	4
Motivation (5)	[50, 51, 56, 105, 125]	3	2	1	1	1				5	3
Knowledge of SDM, policy (4)	[29, 58, 105, 118]	4		2		1				7	
Satisfied with current DM approach (3)	[105, 107, 124]	1		1		2				4	
Characteristics of the adopters (59)											
Child/parent health status (17)	[38, 43, 44, 46, 48, 50, 51, 54, 56–59, 64, 65, 69, 70, 75, 87, 90, 99, 101]	3	3	5	3	3	3		1	11	10
Parent/child’s emotional state (16)	[29, 34, 38, 41, 46, 55, 64, 66, 67, 77, 84, 87, 93, 95, 96, 99]	5	5	5	1	3		1		14	6
Child’s age and competence (15)	[29, 37, 38, 42–44, 48, 52–54, 70, 86, 87, 96, 99]	4	6		1	4	2			8	9
HCP’s SDM skills (14)	[29, 64, 66, 68, 69, 75, 95, 97, 99–101, 108, 116, 124]	3	3		1	2	1	5		10	5
Parent/child race, ethnicity, culture, and language (7)	[58, 67, 89, 93, 95, 103, 125]	3	2	2				1	1	6	2
Parent socioeconomic status (7)	[88–90, 93, 95, 104, 118]		2	1	3				1	1	6
HCP age/seniority/specialty (6)	[57, 58, 61, 73, 90, 123]	1	3			1		1		3	3
Child’s behavior/maturity (6)	[58, 67, 68, 79, 107, 119]		3	2	1	1				3	4
Parent’s health insurance (5)	[54, 59, 89, 93, 95, 109]	1	1	2				1		4	1
HCPs role as advocate (6)	[60, 73, 91, 111, 113, 115]	1	3		2		1			1	6
Child experience with condition (4)	[67, 69, 72, 104]		2				2				4
HCP assuming parent/child preference for involvement (3)	[61, 85, 112]	1		1		1				3	
Parental absence during SDM discussion (2)	[73, 110]						2				2
Parent health literacy (2)	[94, 125]	1		2						3	
Parent’s sex or gender (2)	[108, 123]				1				1		2
Totals		39	44	29	33	28	25	9	4	111	108
Relational level (i.e., social influences) (49)		B	F	B	F	B	F	B	F	B	F
Trust and respect in relationship (29)	[30, 34, 41, 46, 49, 50, 52, 55, 61, 63–68, 71, 72, 75, 80, 82, 84, 87–89, 92, 96, 99, 104, 105]	2	7	2	13	1	7		1	5	28
Extent adopters invite/support parent/child participation in DM (23)	[29, 60, 66, 69–71, 75, 83, 92, 94, 96, 98, 102, 107–109, 111, 112, 115–117, 123, 124]	1	4	3	8	1	4	2	1	7	17
Power relations (17)	[66, 68–70, 73, 81, 87, 96, 106, 107, 110, 111, 114, 116, 119, 121]	3		3	1	9	1	1		16	2
Biasing other adopters (12)	[58, 59, 64, 74, 97, 99, 104, 109, 119, 120]	5		1		2		3		11	
Recognition of HCP/parent expertise (6)	[72, 81, 86, 87, 112, 124]		1	1	4	1				2	5
Conflict (3)	[64, 116, 119]	1		2		1				4	
Totals		12	12	12	26	15	12	6	2	45	52
Environmental level (37)		B	F	B	F	B	F	B	F	B	F
Time (11)	[29, 62–64, 69, 105, 107, 109, 119, 124, 125]	8	1	2		2				12	1
Access to tools/resources/training to promote SDM (10)	[52, 53, 55, 56, 65, 67, 82, 103, 104, 122, 125]	4	6		2		1			4	10
Workflow and continuity of care (10)	[63, 69, 84, 104, 105, 109, 115–117, 125]	8	2	2		1				11	2
Norms (e.g., organizational policy consistent with SDM, expectations that HCP make the decision) (11)	[29, 64, 68, 74, 94, 104, 105, 107, 113, 126]	5	2	3	1	4	1			12	4
Clinical setting (e.g., emergency room) or situation (e.g., urgency) (8)	[29, 58, 87, 104, 105, 107, 114, 127]	4	2	3	1	1				8	3
Physical arrangement (e.g., seating) (3)	[96, 104, 124]	1				1			1	2	1
Stability of home environment (2)	[59, 119]	1		1			1			2	1
Totals		31	13	11	4	9	3		1	50	22

*Qual qualitative, *Quant* quantitative, *MM* mixed methods, *B* barrier, *F* facilitator, *HCP* healthcare provider, *SDM* shared decision-making

#### Decision level (*n* = 19 studies)

##### Barriers

Features of the options was the most frequently cited barrier category at the level of the decision (Table 4), was reported by all adopters, and was the main barrier reported by parents. Features included a perceived lack of options, unacceptable alternatives, and affordability. Adopters, particularly parents, also reported that lack of research evidence for the various options was a barrier to engaging in the SDM process.

##### Facilitators

The perceived magnitude of the decision being discussed influenced the extent to which SDM was encouraged and preferred. Overall, lower stake decisions were reported by all adopters to facilitate SDM in pediatrics. Specifically, HCPs and parents reported being more willing to involve children in decisions when the potential outcomes were considered less risky. Similarly, children reportedly preferred to be involved in lower stake decisions.

#### Innovation level (i.e., SDM; *n* = 34 studies)

##### Barriers

All participant types reported that poor quality information about the condition and/or options that were inappropriately tailored to the child and family’s health literacy needs hindered SDM (Table 4). Additionally, HCPs reported that engaging in the SDM process required too much time and, therefore, lacked feasibility in the pediatric clinical setting.

##### Facilitators

The most commonly cited facilitator for pediatric SDM was high-quality information that was appropriately tailored to the child’s developmental needs and the child/parent literacy needs (e.g., provided in lay terms). High-quality information included the presentation of options, their associated risks and benefits, and research evidence. Some HCPs and children also reported the potential for SDM to improve the way time was used in the clinical encounter.

#### Adopter level (i.e., HCPs, parents, children; *n* = 70 studies)

##### Barriers

Parent’s and child’s emotional state was the most commonly reported barrier at the adopter level (Table 4). This was described to hinder the SDM process when the parent and/or child felt overwhelmed, anxious, in denial, or defensive. Similarly, perceptions of poorer health status of the parent and/or child affected whether they were included, or wanted to be included, in decision-making. Some studies showed that children lacked agreement with SDM in principle and did not prefer SDM to traditional (patriarchal) decision-making approaches. Often HCPs lacked SDM skills, such as knowing how or when to elicit and incorporate family values and preferences in the decision-making process. Lack of HCP skill for SDM was the most frequently cited barrier reported by observers.

##### Facilitators

Agreement with, and desire for, a SDM approach was the most commonly reported facilitator at the adopter level, reported by all adopters (Table 4), and was particularly important to parents. Adopters thought that SDM was the “right thing to do,” that parent and child involvement was important, and that SDM would improve patient outcomes (e.g., satisfaction with the decision-making process). When parents and/or children were in good health, it facilitated efforts to include them in SDM as well as parent/child preference for participation. More efforts were made to include children who were older and perceived to have adequate decision-making competence, particularly among HCPs.

#### Relational level (*n* = 49 studies)

##### Barriers

Power imbalance was the most cited relational barrier, and the most frequently cited by children (Table 4). Power imbalances were described as the systematic exclusion of children from the decision-making conversation or the child feeling too disempowered or intimidated to partner in SDM discussions. All participant types reported that deliberately biasing the opinion of another undermined the SDM process. This was often characterized as the HCP providing only one option, providing information on his or her preferred options only, using SDM to achieve compliance for his or her preferred option, or giving a specific recommendation.

##### Facilitators

Trust and respect in relationships between adopters, primarily between HCPs and family, was a highly cited facilitator, and particularly important for parents. This was characterized by positive relationships, respectful communication, appreciation for each adopter’s expertise, trusting that children will participate in meaningful ways, and that adopters will be open and forthcoming. All participant types reported that inviting and supporting the child and family throughout the SDM process was a facilitator.

#### Environment level (i.e., pediatric clinical practice; *n* = 37 studies)

##### Barriers

Insufficient time due to heavy workloads was the main environmental barrier and the most cited by HCPs. Similarly, clinic workflow (e.g., integrating SDM into the care pathway) and poor continuity of care (e.g., high staff turnover) was reported to hinder SDM. Practice norms, such as the cultural expectation that a HCP’s duty was to provide specific recommendations or make the decision, was a barrier, mostly reported by HCPs and children.

##### Facilitators

The most common environmental facilitator, cited primarily by HCPs, was access to SDM tools (e.g., patient decision aids), resources (e.g., decision coaches or experts in SDM), and/or training.

### Factors influencing pediatric SDM and the OMRU

**Fig. 4:**
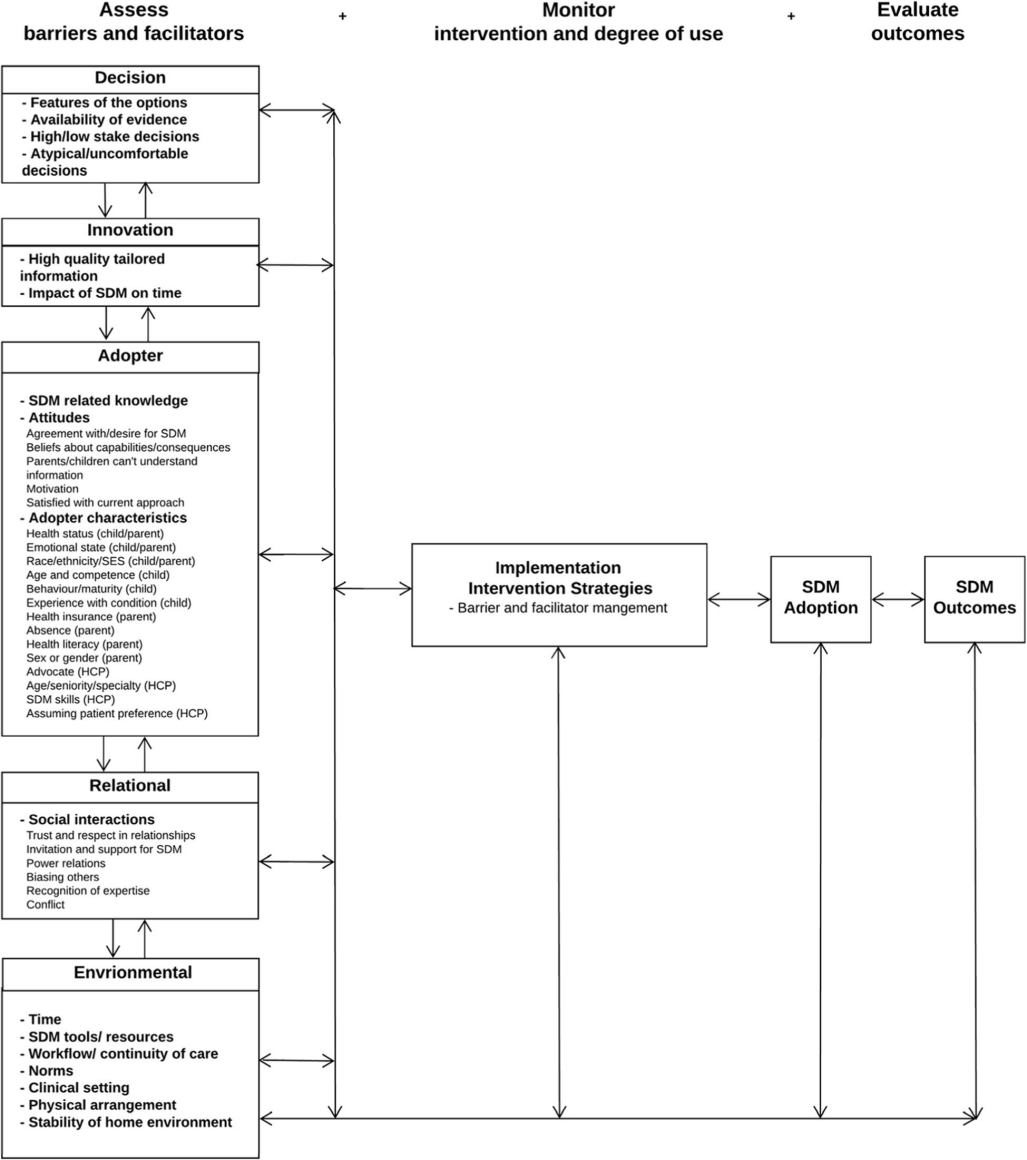
Factors influencing SDM in pediatric clinical practice mapped to the OMRU. Adapted from Logan and Graham, 2010. Double arrows and feedback loops depict the interrelated nature of influential factors existing exist within a system. Influential factors can be present or absent

To illustrate how barriers and facilitators can inform the implementation process, we mapped pediatric SDM influential factors (i.e., not separated into barriers and facilitators) to the OMRU (Fig. 4) [21]. Additionally, we tailored the OMRU for the pediatric SDM context by adding the decision and relational levels. The far left of the figure denotes our results for the assessment of pediatric SDM barriers and facilitators across various levels (i.e., decision, innovation, adopter, relational, and environment). The double arrows show that influencing factors are interrelated and dynamic. According to the OMRU, these influencing factors should inform the development of implementation strategies designed to promote innovation use by minimizing barriers and leveraging facilitators. For example, effective patient decision aids designed for pediatric practice could enhance the high-quality information that is provided to families [5, 13]. The middle column indicates these interventions or strategies should be monitored for impact and degree of use. Finally, the far right of the model shows that interventions should be evaluated for evidence of impact and innovation uptake. Given that the model is iterative, sustained innovation use may require ongoing barrier and facilitator assessments and/or additional implementation strategies (e.g., more of the same implementation strategies or new ones targeting emergent barriers) [21, 30].

## Discussion

We conducted a systematic review of factors influencing pediatric SDM across OMRU levels and from the perspective of HCPs, parents, children, and observers. At each OMRU level, the most frequent barriers were features of the options (decision), poor quality and/or insufficiently tailored information (innovation), parent/child emotional state (adopter), power relations (relational), and insufficient time for SDM (environment). The most frequent facilitators were lower stake decisions (decision), good quality information that is tailored to the families’ literacy and developmental needs (innovation), agreement with SDM (adopter), trust and respect in the relationship (relational), and SDM tools/resources (environment). Across participant types, the most frequently cited barriers were insufficient time (HCPs), features of the options (parents), power imbalances (children), and HCP skill for SDM (observers). The most frequently cited facilitators were good quality information that is tailored to the families’ literacy and developmental needs (HCPs), and agreement with and desire for SDM (parents and children). There was no consistent facilitator category for observers. These findings lead us to make the following observations, which we report at each OMRU level (decision, innovation, adopter, relational, and environment).

At the decision level, antecedent influential factors can impact SDM before the process begins. For example, several adopters felt SDM was unnecessary when they perceived only one reasonable option. Similarly, the perceived magnitude of the decision (high or low stakes) influenced whether adopters attempted to include the child or whether the child wished to participate. To facilitate SDM, HCPs should disclose all reasonable options, including the option of doing nothing [31]. Parents and children reported needing to be invited to engage in SDM for both high and low stake decisions. Additionally, adopters could broaden their conceptualization of SDM from an information exchange process (i.e., HCPs provide the medical evidence and patients relay their preferences) to empowering the patient and family by enabling discussion and participation and providing support for deliberation about the best treatment option [32, 33].

Our review identified few barriers at the innovation level, implying that adopters were generally satisfied with SDM as an innovation when they received high-quality information. However, HCPs reported that SDM took too much time. A Cochrane review that examined patient decision aids for supporting SDM found limited evidence that this SDM intervention took more time [13]. A pilot study evaluating decision coaching using a patient decision aid to prepare children and parents for SDM with their physician took a median of 35 min [34], though the time subsequently spent with the physician was not measured. Given the widespread perceptions about insufficient time for SDM, implementation interventions could emphasize that time spent on SDM is time spent differently, with the potential to increase downstream efficiencies, treatment adherence, and build decision-making capacity in children [35].

At the adopter level, our study showed that most HCPs, parents, and children had positive attitudes about SDM, recognizing that SDM led to positive outcomes for children and families and potentially the system. Nonetheless, some children expressed uncertainty about SDM’s utility and preferred to avoid the burden of decision-making. HCPs and parents often assumed that younger children were unable to participate in SDM, and several HCPs reported that parents and children could not sufficiently understand the medical information needed to engage in decision-making. Yet, observers reported that HCPs lacked the skills needed to translate information and engage families in SDM, limiting parent's and children’s opportunities to get high-quality information. Implementation interventions should emphasize that SDM is a partnership between adopters with shared responsibility for the decision. In contrast to autonomous decision-making, children can be empowered to participate to the extent they are able, through elicitation and consideration of their preferences and views [10]. Typically, informed consent for treatment is a legal requirement, yet unachievable if parents do not understand the information. Our findings suggest that pediatric HCPs require additional education, training, and support to ensure they have the skills to provide families with high-quality information they can understand and use during the SDM process.

At the relational level, our study showed that adopters frequently reported that power imbalances hindered SDM. Adopters recognized that children and parents have difficulty negotiating decision-making involvement and required HCPs’ encouragement and support to participate. A systematic review that evaluated the impact SDM among disadvantaged groups (e.g., low literacy, minority, lower socioeconomic status) found that SDM interventions significantly improved outcomes for vulnerable populations, perhaps more so than individuals in higher literacy and socioeconomic situations [36]. As such other vulnerable groups, such as children, are good candidates for SDM [37]. Consistent with findings from another review that evaluated patient’s perceived barriers to SDM in adult medicine [9], parents and children want to be empowered with an invitation to participate in SDM and high-quality information to enhance their knowledge for decision-making. As parents and children become more knowledgeable about their illness and healthcare system, they report increased capacity for SDM [29]. Our findings showed that an invitation to participate in decision-making should be supported with information that is consistent with the child’s developmental stage and/or the parent’s literacy level. This can be achieved by assessing health literacy levels and tailoring the information accordingly, using child-friendly and developmentally appropriate information, eliciting and incorporating the parent/child’s preferences and values, and verified using teach-back methods [38–41]. Furthermore, trust and respect between HCPs and families can decrease power imbalances by making the parent and child more comfortable asking questions [42].

At the environment level, insufficient time to engage in the SDM process was the most commonly reported barrier, particularly by HCPs. This finding is consistent with HCP reports in another systematic review of SDM barriers in adult medicine [8]. Additionally, workflow and expectations that HCPs make the decision were commonly reported. A recent scoping review of environmental barriers and facilitators to SDM in adult practice recommends countering these predominant barriers by improving the distribution of HCP’s workloads, decreasing pressure for short interactions with patients, and enhancing patient pathway flexibility and scheduling [43]. However, more research is needed to inform changing norms and cultural and societal expectations for SDM.

### Implications and suggestions for future work

The findings of this review suggest that numerous barriers and facilitators influence the implementation of SDM and that each adopter type can experience or perceive different barriers and facilitators. Findings of a Cochrane review suggest that SDM interventions targeting the interprofessional team as well as patients could improve the adoption of SDM in clinical practice [15]. Although few interventions have been evaluated to promote SDM in pediatric clinical practice [5, 6], our review suggests that HCPs, parents, and children would benefit from evidence-based interventions that are specifically tailored to their perceived and/or experienced barriers and facilitators. Consistent with the OMRU, our taxonomy can inform the development of interventions that minimize pediatric SDM barriers and leverage facilitators to improve SDM use. Future research is also needed to examine the nature and strength of the relationships between influential factors to better understand the circumstances in which they interact within the healthcare system to impact SDM use in pediatric clinical practice.

Contextual factors are important for shaping decisions about policy development for health innovations [44]. To promote the uptake of SDM in pediatric clinical practice, decision-makers should consider the influential factors, including those relevant to their unique context, and create policies that aim to minimize barriers and leverage facilitators. For example, organizational policies can foster supportive SDM environments for HCPs, children, and parents [30]. Supportive environments could prioritize patient and family-centered care, partnerships with families, team-based care, decision support, as well as decrease pressure for minimum consultation lengths and incorporate SDM into their clinical practice guidelines and accreditation standards [45–47].

### Strengths and limitations

To our knowledge, this is the first systematic review to focus on the barriers and facilitators of SDM in pediatric clinical practice and is strengthened by the presentation of multiple perspectives (i.e., HCPs, parents, children, and observers) structured using a theoretical implementation model (OMRU). However, several limitations should be considered. Meta-analysis was not possible due to heterogeneity across methodological approaches and measures in the quantitative studies. Therefore, we conducted a narrative synthesis. Due to the large number of included studies with qualitative and quantitative data, we synthesized barriers and facilitators using counting techniques, therefore, not accounting for the effect size. Researcher influence inherently impacts the analysis of qualitative data. At the level of a systematic review, participant-reported data is subject to third reviewer interpretation (those of the original authors and ours), therefore, posing a fidelity risk between the participants’ original statement and our interpretations [48]. Notably, many studies originated from the USA (44%), potentially reflecting barriers and facilitators that are unique to the US healthcare system. For example, features of the options and parental health insurance were often cited by US studies. As such, not all influential factors are relevant or applicable to all contexts. Although we critically appraised included studies and reported potential sources of bias (overall and individually), we did not perform a sensitivity analysis. As such, it is possible that highly biased evidence was given undue weight and low biased studies were underemphasized [49]. Finally, our search was conducted in 2017. Given that our review included 79 studies, it is less likely that newly published studies will have a significant impact on our findings.

### Conclusions

Our study synthesized the barriers and facilitators of implementing SDM in pediatric practice. Pediatric SDM is gaining interest and momentum among researchers with an increasing number of relevant publications each year. Indeed, numerous and diverse barriers and facilitators influence HCPs, parents, and children’s ability to use SDM in pediatric clinical practice. Our study provides a foundation for improved understanding of the factors influencing pediatric SDM use and how to manage them. Future research can use our taxonomy to inform the selection, tailoring, and/or development of knowledge translation interventions to promote SDM in pediatric clinical practice. Policy makers should also consider the context influencing SDM use in pediatrics to reduce barriers and leverage facilitators. Such efforts could improve the health of children by supporting and empowering them to engage in SDM and make high-quality decisions that are consistent with their informed values and preferences.

## Additional file

Additional file 1: CINAHL electronic search. (DOCX 133 kb)
