# Supplementary material for: Barriers and facilitators of pediatric shared decision-making: a systematic review
Source: Implement Sci. 2019 Jan 18;14:7. doi: 10.1186/s13012-018-0851-5 (PMC6339273; doi:10.1186/s13012-018-0851-5)
Supplement: Supplementary file 1 — CINAHL electronic search. (DOCX 133 kb) [file 13012_2018_851_MOESM1_ESM.docx]

Additional file 1 - Search strategy for CINAHL

| # | Query | Results |
| --- | --- | --- |
| **S57** | **S52 AND S56** | **2,657** |
| **S56** | **S53 OR S54 OR S55** | **492,243** |
| S55 | TI ( (infant* or infancy or newborn* or baby or babies or child or children or schoolchild* or "school age" or preschool* or toddler* or adoles* or teen* or boy* or girl* or minors or pediatric* or paediatric*or "nursery school" or kindergar* or "primary school" or "secondary school" or "elementary school" or "high school" or highschool)) OR AB ( (infant* or infancy or newborn* or baby or babies or child or children or schoolchild* or school age*or preschool* or toddler* or adoles* or teen* or boy* or girl* or minors or pediatric* or paediatric*or "nursery school" or kindergar* or "primary school" or "secondary school" or "elementary school" or "high school" or highschool ) ) | 274,949 |
| S54 | (MH "Pediatrics+") | 7,810 |
| S53 | (MH "Child+") OR (MH "Infant+") OR (MH "Minors (Legal)") OR (MH "Adolescence+") | 429,793 |
| **S52** | **S6 OR S49 OR S50 OR S51** | **13,895** |
| **S51** | **S48 AND (S8 OR S9)** | **90** |
| **S50** | **S14 AND S39** | **7,924** |
| **S49** | **S14 AND S31** | **4,549** |
| **S48** | **S40 OR S41 OR S42 OR S43 OR S44 OR S45 OR S46 OR S47** | **66,091** |
| S47 | TI clinician* N4 attitude* OR AB clinician* N4 attitude* OR TI clinician* N4 knowledge OR AB clinician* N4 knowledge OR TI clinician* N4 perspective* OR AB clinician* N4 perspective* OR TI clinician* N4 perception* OR AB clinician* N4 perception* | 1,236 |
| S46 | TI professional* N4 attitude* OR AB professional* N4 attitude* OR TI professional* N4 knowledge OR AB professional* N4 knowledge OR TI professional* N4 perspective* OR AB professional* N4 perspective* OR TI professional* N4 perception* OR AB professional* N4 perception* | 5,850 |
| S45 | TI nurse* N4 attitude* OR AB nurse* N4 attitude* OR TI nurse* N4 knowledge OR AB nurse* N4 knowledge OR TI nurse* N4 perspective* OR AB nurse* N4 perspective* OR TI nurse* N4 perception* OR AB nurse* N4 perception* | 13,488 |
| S44 | TI resident* N4 attitude* OR AB resident* N4 attitude* OR TI resident* N4 knowledge OR AB resident* N4 knowledge OR TI resident* N4 perspective* OR AB resident* N4 perspective* OR TI resident* N4 perception* OR AB resident* N4 perception* | 1,190 |
| S43 | TI doctor* N4 attitude* OR AB doctor* N4 attitude* OR TI doctor* N4 knowledge OR AB doctor* N4 knowledge OR TI doctor* N4 perspective* OR AB doctor* N4 perspective* OR TI doctor* N4 perception* OR AB doctor* N4 perception* | 829 |
| S42 | TI physician* N4 attitude* OR AB physician* N4 attitude* OR TI physician* N4 knowledge OR AB physician* N4 knowledge OR TI physician* N4 perspective* OR AB physician* N4 perspective* OR TI physician* N4 perception* OR AB physician* N4 perception* | 3,091 |
| S41 | TI provider* N4 attitude* OR AB provider* N4 attitude* OR TI provider* N4 knowledge OR AB provider* N4 knowledge OR TI provider* N4 perspective* OR AB provider* N4 perspective* OR TI provider* N4 perception* OR AB provider* N4 perception* | 2,361 |
| S40 | (MH "Attitude of Health Personnel+") | 49,045 |
| **S39** | **S32 OR S33 OR S34 OR S35 OR S36 OR S37 OR S38** | **97,913** |
| S38 | TI caregiver* N2 clinician* OR AB caregiver* N2 clinician* OR TI caregiver* N2 provider* OR AB caregiver* N2 provider* OR TI caregiver* N2 physician* OR AB caregiver* N2 physician* OR TI caregiver* N2 doctor* OR AB caregiver* N2 doctor* OR TI caregiver* N2 nurse* OR AB caregiver* N2 nurse* | 965 |
| S37 | TI families N2 clinician* OR AB families N2 clinician* OR TI families N2 provider* OR AB families N2 provider* OR TI families N2 physician* OR AB families N2 physician* OR TI families N2 doctor* OR AB families N2 doctor* OR TI families N2 nurse* OR AB families N2 nurse* | 1,566 |
| S36 | TI family N2 clinician* OR AB family N2 clinician* OR TI family N2 provider* OR AB family N2 provider* OR TI family N2 physician* OR AB family N2 physician* OR TI family N2 doctor* OR AB family N2 doctor* OR TI family N2 nurse* OR AB family N2 nurse* | 6,399 |
| S35 | TI parent* N2 clinician* OR AB parent* N2 clinician* OR TI parent* N2 provider* OR AB parent* N2 provider* OR TI parent* N2 physician* OR AB parent* N2 physician* OR TI parent* N2 doctor* OR AB parent* N2 doctor* OR TI parent* N2 nurse* OR AB parent* N2 nurse* | 2,074 |
| S34 | TI child* N2 clinician* OR AB child* N2 clinician* OR TI child* N2 provider* OR AB child* N2 provider* OR TI child* N2 physician* OR AB child* N2 physician* OR TI child* N2 doctor* OR AB child* N2 doctor* OR TI child* N2 nurse* OR AB child* N2 nurse* OR TI child* N2 professional* OR AB child* N2 professional* | 4,273 |
| S33 | TI patient* N2 clinician* OR AB patient* N2 clinician* OR TI patient* N2 provider* OR AB patient* N2 provider* OR TI patient* N2 physician* OR AB patient* N2 physician* OR TI patient* N2 doctor* OR AB patient* N2 doctor* OR TI patient* N2 nurse* OR AB patient* N2 nurse* OR TI patient* N2 professional* OR AB patient* N2 professional* | 32,821 |
| S32 | (MH "Professional-Family Relations") OR (MH "Professional-Patient Relations") OR (MH "Nurse-Patient Relations") OR (MH "Physician-Patient Relations") | 61,653 |
| **S31** | **S15 OR S16 OR S17 OR S18 OR S19 OR S20 OR S21 OR S22 OR S23 OR S24 OR S25 OR S26 OR S27 OR S28 OR S29 OR S30** | **43,067** |
| S30 | TI ( caregiver* and participat* ) OR TI ( caregiver* and involv* ) OR TI ( caregiver* and collaborat* ) OR TI ( caregiver* and partner* ) OR TI ( consumer* and participat* ) OR TI ( consumer* and involv* ) OR TI ( consumer* and collaborat* ) OR TI ( consumer* and partner* ) | 516 |
| S29 | TI ( parent* and participat* ) OR TI ( parent* and involv* ) OR TI ( parent* and collaborat* ) OR TI ( parent* and partner* ) OR TI ( relative* and participat* ) OR TI ( relative* and involv* ) OR TI ( relative* and collaborat* ) OR TI ( relative* and partner* ) | 995 |
| S28 | TI ( family and participat* ) OR TI ( family and involv* ) OR TI ( family and collaborat* ) OR TI ( family and partner* ) OR TI ( families and participat* ) OR TI ( families and involv* ) OR TI ( families and collaborat* ) OR TI ( families and partner* ) | 1,313 |
| S27 | TI ( child* and participat* ) OR TI ( child* and involv* ) OR TI ( child* and collaborat* ) OR TI ( child* and partner* ) OR TI ( patient and participat* ) OR TI ( patient and involv* ) OR TI ( patient and collaborat* ) OR TI ( patient and partner* ) | 3,942 |
| S26 | TI consumer* N2 participat* OR AB consumer* N2 participat* OR TI consumer* N2 involv* OR AB consumer* N2 involv* OR TI consumer* N2 collaborat* OR AB consumer* N2 collaborat* OR TI consumer* N2 partner* OR AB consumer* N2 partner* | 659 |
| S25 | TI caregiver* N2 participat* OR AB caregiver* N2 participat* OR TI caregiver* N2 involv* OR AB caregiver* N2 involv* OR TI caregiver* N2 collaborat* OR AB caregiver* N2 collaborat* OR TI caregiver* N2 partner* OR AB caregiver* N2 partner* | 867 |
| S24 | TI relative* N2 participat* OR AB relative* N2 participat* OR TI relative* N2 involv* OR AB relative* N2 involv* OR TI relative* N2 collaborat* OR AB relative* N2 collaborat* OR TI relative* N2 partner* OR AB relative* N2 partner* | 500 |
| S23 | TI parent* N2 participat* OR AB parent* N2 participat* OR TI parent* N2 involv* OR AB parent* N2 involv* OR TI parent* N2 collaborat* OR AB parent* N2 collaborat* OR TI parent* N2 partner* OR AB parent* N2 partner* | 2,882 |
| S22 | TI families N2 participat* OR AB families N2 participat* OR TI families N2 involv* OR AB families N2 involv* OR TI families N2 collaborat* OR AB families N2 collaborat* OR TI families N2 partner* OR AB families N2 partner* | 1,378 |
| S21 | TI family N2 participat* OR AB family N2 participat* OR TI family N2 involv* OR AB family N2 involv* OR TI family N2 collaborat* OR AB family N2 collaborat* OR TI family N2 partner* OR AB family N2 partner* | 3,109 |
| S20 | TI child N2 participat* OR AB child N2 participat* OR TI child N2 involv* OR AB child N2 involv* OR TI child N2 collaborat* OR AB child N2 collaborat* OR TI child N2 partner* OR AB child N2 partner* | 1,139 |
| S19 | TI patient N2 participat* OR AB patient N2 participat* OR TI patient N2 involv* OR AB patient N2 involv* OR TI patient N2 collaborat* OR AB patient N2 collaborat* OR TI patient N2 partner* OR AB patient N2 partner* | 4,305 |
| S18 | (MH "Family Centered Care") | 4,561 |
| S17 | TI patient centered OR AB patient centred OR TI family centered OR AB family centred | 3,609 |
| S16 | (MH "Patient Centered Care") | 13,856 |
| S15 | (MH "Consumer Participation") | 10,250 |
| **S14** | **S7 OR S8 OR S9 OR S10 OR S11 OR S12 OR S13** | **54,106** |
| S13 | TI decision* N1 make OR AB decision* N1 make OR TI decision* N1 making OR AB decision* N1 making | 26,364 |
| S12 | TI decisionmaking OR AB decisionmaking | 334 |
| S11 | TI decision support OR AB decision support | 1,941 |
| S10 | (MH "Decision Support Systems, Clinical") | 1,617 |
| S9 | TI decision aid* OR AB decision aid* | 575 |
| S8 | (MH "Decision Support Techniques") | 1,760 |
| S7 | (MH "Decision Making") OR (MH "Decision Making, Family") OR (MH "Decision Making, Patient") | 32,279 |
| **S6** | **S1 OR S2 OR S3 OR S4 OR S5** | **4,578** |
| S5 | TI ( (share* or sharing or informed or collaborative) ) AND TI ( (decision* or choice*) ) | 1,181 |
| S4 | (collaborative N2 decid*) OR (collaborative N2 decision*) OR (collaborative N2 choice) | 168 |
| S3 | (informed N2 decid*) OR (informed N2 decision*) OR (informed N2 choice*) | 2,916 |
| S2 | (sharing N2 decid*) OR (sharing N2 decision*) OR (sharing N2 choice) | 107 |
| S1 | (share* N2 decid*) OR (share* N2 decision*) OR (share* N2 choice) | 1,420 |
